# The effect of cognitive behavioral therapy text messages on mood: A micro-randomized trial

**DOI:** 10.1371/journal.pdig.0000449

**Published:** 2024-02-21

**Authors:** Marvyn R. Arévalo Avalos, Jing Xu, Caroline Astrid Figueroa, Alein Y. Haro-Ramos, Bibhas Chakraborty, Adrian Aguilera

**Affiliations:** 1 School of Social Welfare, University of California Berkeley, Berkeley, California, United States of America; 2 Centre for Quantitative Medicine, Duke-NUS Medical School, National University of Singapore, Singapore; 3 Faculty of Technology, Policy, and Management, Delft Technical University, Delft, The Netherlands; 4 School of Public Health, Health Policy and Management, University of California Berkeley, Berkeley, California, United States of America; 5 Department of Statistics and Data Science, National University of Singapore, Singapore; 6 Department of Biostatistics and Bioinformatics, Duke University, United States of America; 7 Department of Psychiatry and Behavioral Sciences, University of California–San Francisco, San Francisco, California, United States of America; Iran University of Medical Sciences, IRAN (ISLAMIC REPUBLIC OF)

## Abstract

The StayWell at Home intervention, a 60-day text-messaging program based on Cognitive Behavioral Therapy (CBT) principles, was developed to help adults cope with the adverse effects of the global pandemic. Participants in StayWell at Home were found to show reduced depressive and anxiety symptoms after participation. However, it remains unclear whether the intervention improved mood and which intervention components were most effective at improving user mood during the pandemic. Thus, utilizing a micro-randomized trial (MRT) design, we examined two intervention components to inform the mechanisms of action that improve mood: 1) text messages delivering CBT-informed coping strategies (i.e., behavioral activation, other coping skills, or social support); 2) time at which messages were sent. Data from two independent trials of StayWell are included in this paper. The first trial included 303 adults aged 18 or older, and the second included 266 adults aged 18 or older. Participants were recruited via online platforms (e.g., Facebook ads) and partnerships with community-based agencies aiming to reach diverse populations, including low-income individuals and people of color. The results of this paper indicate that participating in the program improved and sustained self-reported mood ratings among participants. We did not find significant differences between the type of message delivered and mood ratings. On the other hand, the results from Phase 1 indicated that delivering any type of message in the 3 pm-6 pm time window improved mood significantly over sending a message in the 9 am-12 pm time window. The StayWell at Home program increases in mood ratings appeared more pronounced during the first two to three weeks of the intervention and were maintained for the remainder of the study period. The current paper provides evidence that low-burden text-message interventions may effectively address behavioral health concerns among diverse communities.

## Author summary

Mobile health (mHealth) interventions have the potential to improve the mental health of vulnerable populations, including low-income individuals and people of color. During the COVID-19 pandemic our team developed the StayWell at Home intervention, a 60-day text-messaging program, to help vulnerable populations cope with the negative effects of the pandemic. Users enrolled in the program received two daily text-messages. One message contained a mental health support tip randomly selected from different message categories (example: social support) and the second message asking users to rate their mood. The messages were sent randomly throughout the day. In this study we examined whether the message category and message timing had an impact on daily mood across the 60-days of the study. We found that participants in the StayWell at Home intervention improved their mood during the first two to three weeks of the text-messaging program and messages delivered in the afternoon, 3 pm to 6 pm, were more effective at improving mood over messages delivered in the morning, 9 am to 12 pm. The results of this study suggest that low-cost and low-burden text-messaging interventions can be used to promote mental health for vulnerable populations.

## Introduction

COVID-19 has been a devastating infectious disease crisis but has also resulted in a mental health calamity. Pandemic anxiety, social distancing, and devastating economic consequences worsened population-level mental health [1]. Since the start of the pandemic, depression and anxiety symptoms have soared, and experts have called for developing widespread psychological support [2]. Due to social distancing mandates during the COVID-19 pandemic, there was an increased interest in leveraging technology for health promotion, and personal mobile phones became crucial for providing psychological support. We can reach individuals from all sociodemographic backgrounds using text messaging, including essential workers, low-income individuals, and ethnic minority groups, who are also more vulnerable to COVID-19 infection and stress [3,4]. Thus, developing and testing mobile health (mHealth) mental health interventions is critical for supporting the mental health of vulnerable individuals.

At the start of the pandemic, our team developed the StayWell at Home intervention, a 60-day text messaging program based on our group’s previous text-messaging cognitive behavioral therapy (CBT) work [5]. Our goal was to help people cope with the uncertainties and lifestyle changes of the global crisis using evidence-based tools. CBT is based on the idea that our thoughts, behaviors, and emotions determine how we feel. In brief, CBT challenges unhelpful thoughts and helps individuals understand the relationship between actions and mood [6]. As a response to the social distance mandates, our team implemented the StayWell at Home intervention via two independent trials. The results of these independent studies indicated participants experienced significant reductions in depressive and anxiety symptoms after 60 days of exposure to the text messages [7,8]. A secondary outcome of the StayWell at Home intervention was daily mood ratings. mHealth interventions using automated text-messaging indicate that delivery of supportive text-messages helps participants monitor and manage their daily mood [9]. Moreover, daily self-reported mood ratings predict depressive symptoms among diverse populations [9,10]. Due to the utility of daily mood ratings, automated mobile mood tracking technology (i.e., Mood 24/7) is being developed to assist clinicians with an accurate and efficient longitudinal assessment of patients’ symptoms [11]. Thus, in the current paper, we conducted a secondary analysis of the StayWell at Home phase one and phase two to examine the impact of the intervention and its components on mood ratings.

This paper aims to examine the trajectories of mood ratings across the 60-day StayWell at Home intervention. Given that the intervention was associated with reductions in depressive symptoms and the link between mood ratings and depressive symptoms, we hypothesized that the intervention would result in improved mood for participants. Based on our prior studies, we do not know which intervention components were most effective at improving health outcomes. Thus, within a micro-randomized trial (MRT), we examine two components of StayWell at Home to inform the mechanisms of action that improve mood: 1) text messages delivering CBT-informed coping strategies (i.e., behavioral activation, other coping skills, or social support) and 2) time at which messages were sent. MRTs help examine the proximal effects of intervention components and can provide empirical data for personalizing digital health interventions [9] and optimizing just-in-time mHealth adaptive interventions (8). MRTs have been used to examine mHealth physical activity and substance use interventions [12–14]. Yet, limited MRTs have examined mental health outcomes, such as mood or those based on mental health theoretical models [15,16]. Thus, in this paper, we include data from two independent trials of StayWell at Home to examine the role of intervention components in improving mood.

## Methods

### Study design phase 1

The StayWell at Home intervention is a low-intensity 60-day text messaging program based on Cognitive Behavioral Therapy (CBT) and designed to help people cope with COVID-19-related stress. During Phase1, we developed two message types: 1. Behavioral activation messages, which provided tangible tips for identifying active and pleasurable activities, and 2. Other Coping Skills messages, or psychoeducation, included recommendations to help with restructuring thoughts, tips about sleep, and self-care. Examples of behavioral activation messages include: *“Identify a 6+ feet apart activity you can do*. *Can you ask someone you care about to go for a 6+ feet apart walk*?*”* and *“Social distancing is hard*, *but you can still keep connected*. *Try setting up a virtual dinner party with your friends*.*”* Examples of other coping skills messages include: *“If your friend told you they were struggling with quarantine*, *what would you say to them to make them feel better*?*”* and *“Be kind to yourself*. *This situation will not last forever*, *You’ve got this*!*”*. Each day participants received two text messages. First, a tip message was randomly selected from the two categories described above and sent daily between 9 am–6 pm followed by a mood-monitoring message three hours later. We designed the messages in English and Spanish to reach diverse populations, particularly monolingual Spanish speakers who are often excluded from digital health interventions [5]. The delivery of text messages and collection of self-reported mood ratings was conducted via a HIPPA-compliant platform, HealthySMS. The University of California Berkeley Institutional Review Board reviewed and approved all study procedures [7,8].

### Data and participants phase 1

Phase one of StayWell at Home included 303 adults 18 years or older (M = 33.3, SD = 11), mostly female (76%), with a mobile phone who speak English (88.4%) and/or Spanish (11.3%). Participants who used an online text-messaging app (e.g., Google Voice) were excluded from the intervention, as this is more prone to online scams and fraud (e.g., individuals creating fake accounts to receive reimbursements). Study recruitment took place between April and December of 2020. This trial was fully remote, and through targeted ads (e.g., via Facebook), we tried to recruit low-income individuals (84.5%) and people of color (51.8%) who are disproportionately impacted by the negative impacts of COVID-19 in the U.S. Interested participants were automatically enrolled in the text-messaging program after completing a baseline Qualtrics survey.

### Micro-randomization phase 1

Every day during the 60-day text-messaging study, treatment allocation was characterized by a full factorial design with a total of two factors representing messages (M) and the time frame (T) when the message was sent. M has two levels (behavioral activation vs. other coping skills), and T has three levels (9 am-12 pm, 12 pm-3 pm, 3 pm- 6 pm). Within the MRT, each participant was re-randomized daily to receive one message based on the combination of M (0.5 probability) and T (0.33 probability). In other words, every participant received a daily behavioral activation message or other coping skills message at different times of the day. Three hours following M delivery, participants received a message asking them to rate their mood on a scale of 1–9, with 9 being the best mood.

### Statistical analysis phase 1

The proximal effects of message type (M = behavioral activation vs. other coping skills) and time window (T = 9 am-12 pm vs. 12 pm-3 pm, 3 pm-6 pm) on the mood rating over the 60 days were investigated using an approach based on the generalized estimating equations (GEE) and intent-to-treat analysis. Specifically, the models were estimated using the weighted and centered least square (WCLS) method for MRT designs [17]. In model 1, we first examined the effect of M on mood rating using constant, linear, quadratic, and linear plateau trends over the 60 days of the study. Then, in model 2, we examined the effects of T on mood rating using the constant, linear, quadratic, and linear plateau trends.

### Study design phase 2

The Phase 2 StayWell at Home intervention and implementation procedures are similar to those reported earlier in Phase 1. However, we made two modifications; first, we included a new message category representing Social Support tips and an option for not sending a tip message [8]. Social support tips examples include: *“Taking the time to share your feelings and listen and support others will go a long way during the pandemic*, *especially during the holidays”* and *“The positive social contacts you have had today can help you tomorrow*. *Who can you reconnect with today*?*”*

### Data and participants phase 2

Participants in Phase 2 included 266 adults 18 years or older (M = 35.7, SD = 12.4), mostly female (80.5%), and mostly English speakers (85.3%) vs. Spanish speakers (14.7%). Recruitment methods varied slightly, 47% of the sample was recruited via the online (e.g., via Facebook) targeted ads, and the remaining 53% was recruited via partnerships/listservs with community agencies with the goal of using this community-based sampling to increase the representation of Latinx-identified adults. The final sample includes 60% low-income individuals and 64% people of color. For this trial, recruitment took place between February to June 2021.

### Micro-randomization Phase 2

StayWell Phase 2 included four levels of M (behavioral activation, other coping skills, social support, and no message sent), and T had three levels (9 am-12 pm, 12 pm-3 pm, 3 pm- 6 pm). Within the MRT, each participant was re-randomized daily to receive one message based on the combination of M (0.25 probability) and T (0.33 probability). Self-reported mood rating (range 1–9, 9 being best) was collected three hours after the delivery of M.

### Statistical Analysis Phase 2

We replicated the analysis described earlier and examined the proximal effects of message type (M = none vs. behavioral activation, other coping skills, and social support) and time window (T = 9 am-12 pm vs. 12 pm-3 pm, 3 pm-6 pm) on the mood rating over the 60-day study period.

## Results

### Phase 1 results

This MRT examined the proximal effects of message type (M–two levels) and time frame (T–three levels) on mood rating. For our first model, testing M on mood, the results indicated no statistically significant (P > .05) difference in effect between the behavioral activation and the other coping skills type of messages on mood (3 hours after a message). These findings were robust to the choice of constant, linear, quadratic, and linear plateau trends (Table 1). For our second model, testing T on mood, the results indicated that based on a constant trend, messages sent during the 3 pm-6 pm time window significantly increased the mood rating (b = 0.1806, P < .001) relative to messages sent during the 9 am-12 pm time window (Table 2).

**Table 1:** The generalized estimating equation model: estimating the message type proximal effects on mood rating under a constant, linear, quadratic and linear-plateau trend of study days respectively, StayWell Phase 1 (N = 303).

Trend	Predictor	Estimate	Standard Error	P-value
Constant	Intercept	6.5092	0.0891	< 0.001
	Message-BA Centered	0.0280	0.0387	0.468
Linear	Intercept	6.3657	0.0811	< 0.001
	Day	0.0054	0.0018	0.0021
	Message-BA Centered	0.0882	0.0665	0.1845
	Message-BA Centered*Day	-0.0024	0.0021	0.2504
Quadratic	Intercept	6.2444	0.0834	< 0.001
	Day	0.0194	0.0050	< 0.001
	Day^2^	-0.0003	0.0001	0.0038
	Message-BA Centered	0.0816	0.1023	0.4251
	Message-BA Centered*Day	-0.0017	0.0088	0.8481
	Message-BA Centered*Day^2^	0.0000	0.0002	0.9457
Linear-Plateau	Intercept	6.2403	0.0829	< 0.001
	*Min*(Day, 21)	0.0172	0.0042	< 0.001
	Message-BA Centered	0.1020	0.0961	0.2888
	Message-BA Centered*Min(Day, 21)	-0.0048	0.0056	0.3928

Note that ‘Message-BA’ is a dummy variable of BA message type while ‘Centered’ represents minus the randomization probability of message type, i.e., ½. ‘*Min*’ indicates minimum function.

**Table 2:** The generalized estimating equation model: estimating the time window proximal effects on mood rating under a constant, linear, quadratic and linear-plateau trend of study days respectively, StayWell Phase 1 (N = 303).

Trend	Predictor	Estimate	Standard Error	P-value
Constant	Intercept	6.5144	0.0889	< 0.001
	Time-12-3 pm Centered	0.0176	0.0416	0.6726
	Time- 3–6 pm Centered	0.1806	0.0502	0.0003
Linear	Intercept	6.3703	0.0810	< 0.001
	Day	0.0054	0.0018	0.0020
	Time-12-3pm Centered	-0.0451	0.0746	0.5456
	Time-3-6 pm Centered	0.0923	0.0830	0.2658
	Time-12-3pm Centered*Day	0.0022	0.0024	0.3571
	Time-3-6 pm Centered*Day	0.0032	0.0026	0.2224
Quadratic	Intercept	6.2449	0.0839	< 0.001
	Day	0.0198	0.0050	< 0.001
	Day^2^	-0.0003	0.0001	0.0028
	Time-12-3pm Centered	-0.0852	0.1057	0.4202
	Time-3-6 pm Centered	-0.0259	0.1147	0.8213
	Time-12-3pm Centered*Day	0.0069	0.0096	0.4708
	Time-3-6 pm Centered*Day	0.0163	0.0098	0.0961
	Time-12-3pm Centered*Day^2^	-0.0001	0.0002	0.6271
	Time-3-6 pm Centered*Day^2^	-0.0002	0.0002	0.1854
Linear-Plateau	Intercept	6.2426	0.0830	< 0.001
	*Min*(Day, 21)	0.0173	0.0041	< 0.001
	Time-12-3pm Centered	-0.1030	0.1027	0.3156
	Time-3-6 pm Centered	-0.0087	0.1118	0.9376
	Time-12-3pm Centered*Min(Day, 21)	0.0076	0.0062	0.2192
	Time-3-6 pm Centered*Min(Day, 21)	0.0118	0.0064	0.0663

Note that ‘Time-12-3pm’ is a dummy variable of time window during 12-3pm while ‘Centered’ represents minus the randomization probability of time window, i.e., 1/3. ‘*Min*’ indicates minimum function.

The results of this analysis also provide evidence on mood rating trends across time (i.e., 60 days of intervention) independent of M and T. Both Tables 1 and 2 present the constant, linear, quadratic, and linear plateau mood rating trends. For a constant trend, the average mood rating over the study period was 6.51 (P < .001). For a linear trend, the mood rating was 6.37 (P < .001) on day one and increased with a daily rate of 0.0054 (P = .002). For a quadratic trend, there was a significant quadratic effect of intervention day (b = -0.0003, P = .004). The mood rating was 6.24 (P < .001) on day one, increased initially but decreased after 5 weeks. For a linear plateau trend, the mood rating was 6.24 (P < .001) on day one, increased at daily rate 0.017 (P < .001) until day 21, then maintained the maximum value at 6.58.

**Fig 1:**
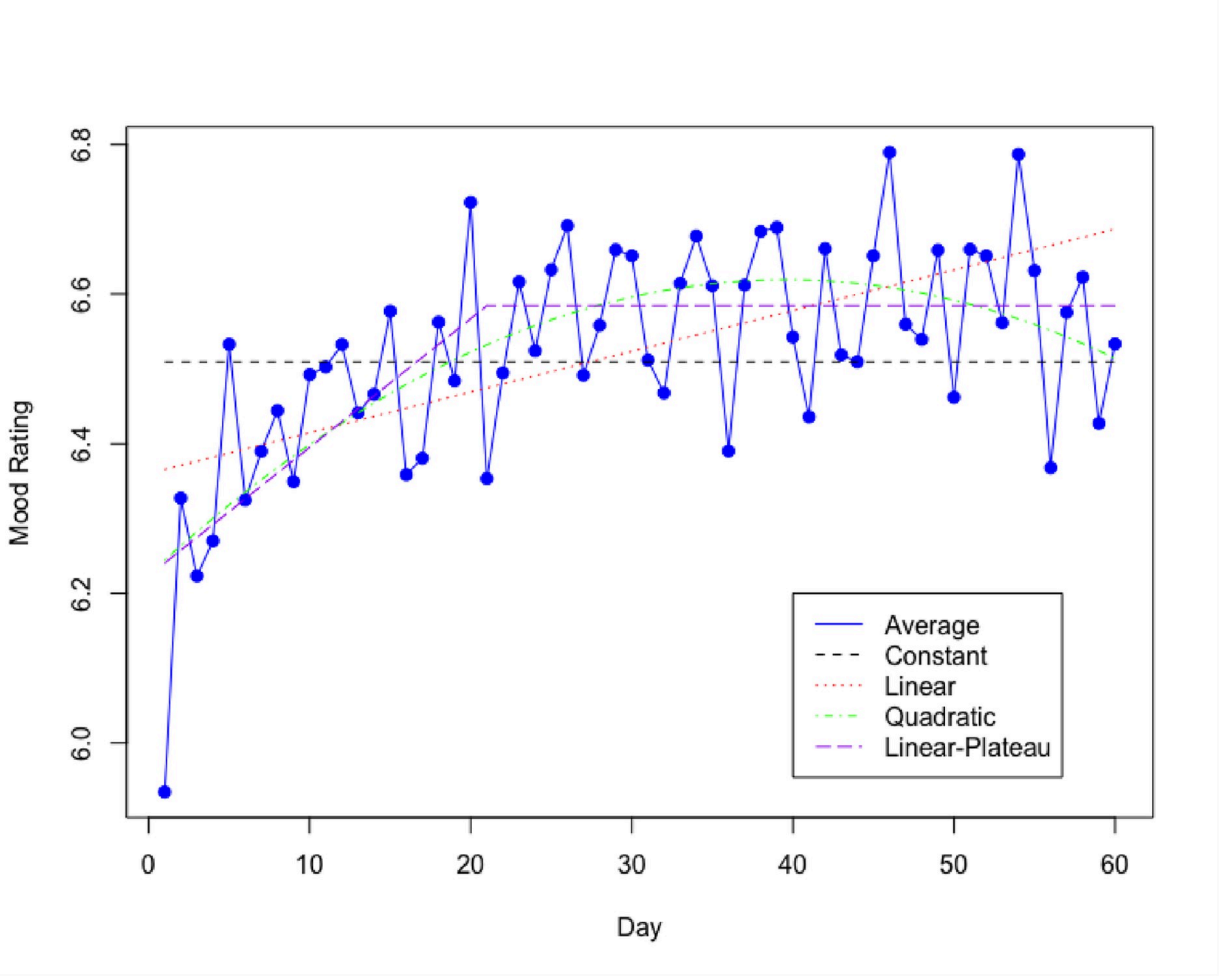
Mood Rating Trends–StayWell Phase 1.

Fig 1 presents the plots of the expected mood rating on each day of the 60-day intervention period. These trends are based on the sample average and the first model tested which included M type (Table 1). The trend plots indicate that the mood rating estimated by the linear-plateau trend provides the best fitting trend for the average mood rating on each day of the intervention. The constant trend does not capture the increasing pattern of the mood rating in the early days of the intervention. From the mid to later days of the intervention the average mood ratings do not show an increasing pattern as indicated by the linear trend. Lastly, in the later days of the intervention, the average mood ratings do not show a decreasing pattern as depicted by the quadratic trend. Thus, the linear-plateau trend, depicting an initial increasing pattern in mood rating before stabilizing, provides the best fitting model of average mood rating.

Lastly, we examined the response rate to the mood rating messages and found that on average participants responded to 60.4% of these messages. We also conducted a sensitivity analysis using the same approaches described above. The results were similar when the missing values mood rating were imputed using multiple imputation (chained equation methods) [18]. For more parsimonious reporting of study results, we have presented only the non-imputed results above.

### Results Phase 2

This MRT examined the proximal effects of message type (M–four levels) and time frame (T–three levels) on mood rating. Table 3 presents the estimated proximal effect of message types on mood. We did not find statistically significant (p>0.05) differences between behavior activation, other skills, social skill, and no message categories under the constant, linear, quadratic, or linear plateau trend. Table 4 presents the estimated proximal effect of time windows on mood. We did not find a significant (p>0.05) difference between the 9 am-12 pm, the 12–3 pm, and the 3–6 pm time windows under the constant, linear, quadratic, or linear plateau trend.

**Table 3:** The generalized estimating equation model: estimating the message type proximal effects on mood rating under a constant, linear, quadratic, and linear-plateau trend of study days, respectively, StayWell Phase 2(N = 266).

Trend	Predictor	Estimate	Standard Error	P-value
Constant	Intercept	6.5614	0.0811	< 0.001
	Message-BA Centered	0.0646	0.0498	0.1945
	Message-Skills Centered	0.0990	0.0539	0.0662
	Message-SS Centered	0.0573	0.0500	0.2526
Linear	Intercept	6.4416	0.0835	< 0.001
	Day-1	0.0044	0.0015	0.0025
	Message-BA Centered	0.0030	0.0968	0.9757
	Message-Skills Centered	0.0697	0.1073	0.5160
	Message-SS Centered	0.1658	0.0942	0.0786
	Message-BA Centered*(Day-1)	0.0025	0.0031	0.4320
	Message-Skills Centered*(Day-1)	0.0012	0.0030	0.6999
	Message-SS Centered*(Day-1)	-0.0039	0.0029	0.1684
Quadratic	Intercept	6.3610	0.0883	< 0.001
	Day-1	0.0137	0.0046	0.0030
	(Day-1)^2^	-0.0002	0.0001	0.0298
	Message-BA Centered	-0.0498	0.1407	0.7232
	Message-Skills Centered	0.1109	0.1560	0.4773
	Message-SS Centered	0.1038	0.1435	0.4694
	Message-BA Centered*(Day-1)	0.0087	0.0121	0.4744
	Message-Skills Centered*(Day-1)	-0.0036	0.0126	0.7715
	Message-SS Centered*(Day-1)	0.0029	0.0118	0.8075
	Message-BA Centered*(Day-1)^2^	-0.0001	0.0002	0.5877
	Message-Skills Centered*(Day-1)^2^	0.0001	0.0002	0.6890
	Message-SS Centered*(Day-1)^2^	-0.0001	0.0002	0.5491
Linear-Plateau	Intercept	6.2840	0.0931	< 0.001
	Min(Day-1, 14–1)	0.0250	0.0060	< 0.001
	Message-BA Centered	-0.0712	0.1620	0.6604
	Message-Skills Centered	0.0659	0.1779	0.7110
	Message-SS Centered	0.1372	0.1708	0.4218
	Message-BA Centered*Min(Day-1, 14–1)	0.0125	0.0141	0.3747
	Message-Skills Centered*Min(Day-1, 14–1)	0.0029	0.0145	0.8397
	Message-SS Centered*Min(Day-1, 14–1)	-0.0074	0.0143	0.6072

Note that ‘Message-BA’ is a dummy variable of BA message type while ‘Centered’ represents minus the randomization probability of message type, i.e., 1/3. ‘*Min*’ indicates minimum function.

**Table 4:** The generalized estimating equation model: estimating the time window proximal effects on mood rating under a constant, linear, quadratic and linear-plateau trend of study days respectively, StayWell Phase 2 (N = 266).

Trend	Predictor	Estimate	Standard Error	P-value
Constant	Intercept	6.5641	0.0816	< 0.001
	Time-12-3pm Centered	-0.0458	0.0413	0.2678
	Time-3-6 pm Centered	0.0239	0.0561	0.6694
Linear	Intercept	6.4490	0.0841	< 0.001
	Day-1	0.0042	0.0015	0.0043
	Time-12-3pm Centered	0.0056	0.0853	0.9473
	Time-3-6 pm Centered	0.0950	0.0895	0.2883
	Time-12-3pm Centered*(Day-1)	-0.0019	0.0027	0.4764
	Time-3-6 pm Centered*(Day-1)	-0.0026	0.0029	0.3643
Quadratic	Intercept	6.3636	0.0891	< 0.001
	Day-1	0.0140	0.0047	0.0027
	(Day-1)^2^	-0.0002	0.0001	0.0244
	Time-12-3pm Centered	0.0264	0.1152	0.8187
	Time-3-6 pm Centered	0.0777	0.1298	0.5495
	Time-12-3pm Centered*(Day-1)	-0.0045	0.0091	0.6210
	Time-3-6 pm Centered*(Day-1)	-0.0008	0.0110	0.9450
	Time-12-3pm Centered*(Day-1)^2^	0.0000	0.0002	0.7625
	Time-3-6 pm Centered*(Day-1)^2^	0.0000	0.0002	0.8555
Linear-Plateau	Intercept	6.2836	0.0936	< 0.001
	*Min*(Day-1, 14–1)	0.0253	0.0061	< 0.001
	Time-12-3pm Centered	0.0738	0.1408	0.6002
	Time-3-6 pm Centered	0.0503	0.1596	0.7525
	Time-12-3pm Centered*Min(Day-1, 14–1)	-0.0110	0.0119	0.3563
	Time-3-6 pm Centered*Min(Day-1, 14–1)	-0.0025	0.0146	0.8615

Note that ‘Time-12-3pm’ is a dummy variable of time window during 12-3pm while ‘Centered’ represents minus the randomization probability of time window, i.e., 1/3. ‘Min’ indicates minimum function.

Tables 3 and 4 present the trends, i.e., constant, linear, quadratic, and linear plateau in terms of days, for mood rating over the study period, regardless of message types and time windows. For a constant trend, the average mood rating over the study period is 6.56 (P < .001). For a linear trend, the mood rating is 6.44 (P < .001) on day one and increases with a daily rate of 0.0044 (P = 0.0025). For a quadratic trend, there is a significant quadratic effect of study day (b = -0.0002, P = 0.0298). The mood rating started at 6.36 (P < .001) on day one, and increased initially; however, the trend began to decrease after five weeks. For a linear-plateau trend, the mood rating is 6.28 (P < .001) on day one, increases at a daily rate of 0.025 (P < .001) until day 14, then maintains the maximum value at 6.61.

**Fig 2:**
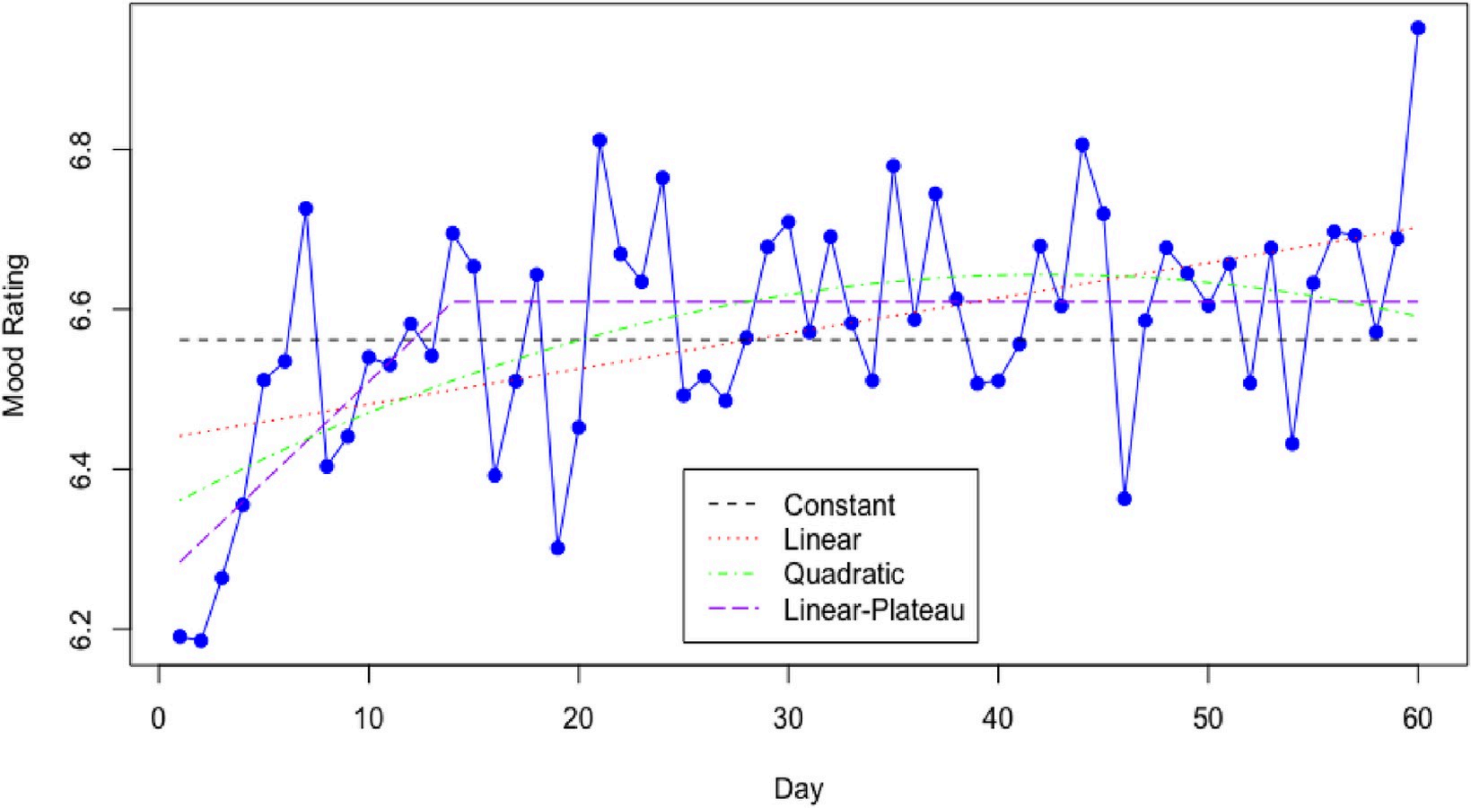
Mood Rating Trends–StayWell Phase 2.

Fig 2 presents the plots of the expected mood rating on each day over the study, based on the sample average and models of message type estimated in Table 3. We observe that the mood rating estimated by the linear plateau trend provides the best fitting for the average of mood rating on each day of the study. The constant trend does not capture the increasing pattern of the mood rating in the early days. From middle to later days, the average mood ratings do not show an increasing pattern captured by the linear trend. In the later days, the average mood ratings do not show an obviously decreasing pattern captured by the quadratic trend.

Similar to phase one, we examined the response rate to the mood rating messages and found that on average participants responded to 59.6% of these messages. The sensitivity analysis using multiple imputations (chained equation methods) [18] for the missing mood ratings resulted in the same findings. For more parsimonious reporting of study results, we have presented only the non-imputed results above.

## Discussion

The purpose of this paper was to conduct a secondary data analysis to examine daily mood trajectories of participants enrolled in a 60-day CBT-based text-messaging intervention, StayWell at Home, and to explore the role of intervention components on mood. We hypothesized that the intervention would result in improved mood ratings and found that across the two phases of the intervention, participants had improved and sustained self-reported mood ratings. StayWell at Home is based on delivering text messages centered on behavioral activation, social support, or other coping skills. Yet, in this MRT, a novel approach for examining the proximal effects of intervention components on the intervention outcomes, we did not find significant differences between the type of message delivered on mood ratings indicating all may be equally important to achieving improvement. On the other hand, results from Phase 1 indicated that delivering any message type in the late afternoon 3 pm- 6 pm time window significantly improved mood compared to sending a message in the 9 am-12 pm window. The StayWell at Home program increases in mood ratings appeared more pronounced during the first two to three weeks of the intervention and were maintained for the remainder of the study period. This is consistent with prior work, finding that after a certain period, participants experience a behavioral response decrement to stimuli [19].

Our prior work with the StayWell at Home program indicated that the intervention was associated with overall reductions in depression and anxiety symptoms as rated by the PHQ and GAD [7,8]. Here, we extend those findings to the outcome of daily mood ratings to better understand continuous symptom fluctuations at the daily level and to develop the evidence of text-messaging programs for supporting mental health outcomes among diverse communities disproportionately affected by the COVID-19 pandemic [4]. By conducting two independent trials during times of heightened COVID-19 stress (e.g., due to quarantines, increased infection rates, and novel COVID strain anxiety) and recruiting nationally representative and diverse samples, we can speculate that the effects of the intervention would be similar for different types of people and in a different context. However, future research may examine the role of contextual factors in response to digital health interventions. A growing body of literature supports mHealth interventions, including text message-based interventions, in promoting mental health outcomes [20]. Yet, these results provide limited information about the accessibility and uptake of mHealth interventions for underserved communities. Thus, it is crucial to continue developing and testing the efficacy of interventions, such as StayWell at Home, which have the potential for widespread dissemination and reach, given the low-intensity format of delivering two text messages daily.

The results on overall mood rating improvements across the study duration are consistent with prior research on mental health interventions in general and mHealth interventions specifically. For example, in a randomized controlled trial, Smyth et al. (2018) found that an online positive affect journaling intervention decreased psychological distress and increased well-being among hospital patients with mild to moderate anxiety. Further, the researchers found these effects were most observed during the first month of the intervention and maintained for 2–3 months later [21]. Sustaining intervention effects over a long period of time has been documented as a limitation and challenge in behavioral health intervention research.

An added value of the StayWell program and this study is that by tracking participants for 60 days we could demonstrate that the initial gain in mood rating was maintained over time. The ongoing, low-intensity delivery of two text messages per day may help individuals stay engaged with the intervention and thus motivated to change over time. It is also likely that participants’ repertoire of coping skills increases as a result of receiving the text message intervention; the increase in coping skills may facilitate greater resilience to stress, and thus the stable mood ratings toward the end of the intervention may indicate that participants have developed some skills to manage stress. Future research would benefit from specifically measuring the application of skills.

Though research findings suggest interventions delivered via text messages are effective at promoting behavior change, there are significant methodological limitations in assessing the efficacy of these interventions [22]. The MRT design of this paper helps provide preliminary data on how the various intervention components influenced intervention outcomes. The results of this paper did not support a difference in the type of message delivered and the mood rating. There are a couple of potential explanations for why we did not find significant effects. First, it is possible that the messages we developed did not fit neatly into either the behavioral activation or the other-coping skills categories. There is a considerable amount of overlap between messages that may encourage behavioral activation versus not. Secondly, it is possible that participants received behavioral activation messages when they were unavailable to act on said recommendations. Last, it is possible that participants received similar benefits from various strategies that are all based on effective treatments for improving mood management and that the effects of one message on mood could carry over to the following days or even weeks. This may help explain overall mood trajectories for Phase 2 participants even when a supportive message was not delivered on a particular day.

On the other hand, this MRT design showed that messages delivered in the afternoon significantly improved mood relative to messages delivered in the morning in Phase 1. There is evidence to suggest that as people go about their day, they accumulate greater stress and deplete their daily resources needed to cope with stress [23]; thus, it may be that the delivery of a message in the afternoon was received better by participants and thus had a more immediate benefit/effect on mood. However, this finding was not replicated in Phase 2, but it would be important to assess this relationship in future studies.

### Implications

The current paper provides evidence that low-burden text-message interventions may effectively address behavioral health concerns among diverse communities. Furthermore, the MRT design allowed for an examination of intervention components, and the results suggested that delivery of text messages in the afternoon may be more beneficial for proximal mood improvements than messages sent in the mid-morning. The analysis also showed an initial improvement in mood that stabilizes after a couple of weeks.

### Limitations

The intervention did not account for differences in participant availability; that is, we needed to find out if some people were unavailable during different times, which would render some of the tips/recommendations ineffective for participants. However, we tried to mitigate this by prompting for mood three hours after the text-message delivery. Following up with participants regarding whether they were available to act on the intervention or developing additional tools for assessing participant availability may help mitigate this concern.

## Conclusion

MRT analyses of mobile mental health interventions can help us understand how just-in-time adaptive interventions work over time. These analyses can show the length and depth of intervention effects and help understand whether certain content or timing is most effective. Based on the MRT design, the results of this paper indicated that mood ratings remain stable over time regardless of the content of the message, yet, messages sent later in the day may have a more direct effect on mood.
